# Surgical repair of large pulmonary artery to left atrium fistula

**DOI:** 10.1002/ccr3.3580

**Published:** 2020-11-24

**Authors:** Alaa Hussain, Muhamed Younis, Samir Srour, Mohamed Saleh, Mohammed Ismael

**Affiliations:** ^1^ Damascus University Cardiovascular Surgical Center Damascus Syrian Arab Republic

**Keywords:** ASD, cyanosis, left atrium, pulmonary artery fistula, surgical repair

## Abstract

We report a successful surgical closure of a rught pulmonary artery to left atrium fistula (RPALAF) with secondary atrial septal defect (ASD) in a 3.5 years old girl. These fistulae are rare cause of cyanosis and require early diagnosis and treatment to prevent the serious complications of them.

## Graphical abstract

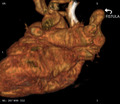

## 1 INTRODUCTION

We report a successful surgical closure of right pulmonary artery to left atrium fistula (RPALAF) with secondary atrial septal defect (ASD) in a 3.5‐year‐old girl, who had presented with central cyanosis and exertional dyspnea. The diagnosis of this anomaly was confirmed by angiography catheterization and multislice computed tomography scanner. The orifice of the fistula in the left atrium was closed by a biological patch. The cyanosis improved directly after surgery.

The pulmonary fistula is an abnormal connection between the pulmonary artery or its branches and the pulmonary veins or left atrium bypassing the pulmonary capillary bed, which causes a right to left shunt and is considered a rare congenital cardiac malformation.^1^ Patients often present with central cyanosis, clubbing, and if treatment is delayed, patients may develop embolic manifestations, cerebrovascular accidents, hyperviscosity symptoms, and heart failure.^1^, ^2^

Pulmonary arteriovenous fistulae are very rare and pulmonary artery to left atrium ones even rarer.^3^, ^4^ This case is difficult to diagnose based solely on clinical and echocardiographic findings. A contrast echo and angiography and/or a multislice CT is needed to establish the structural anatomy of the right pulmonary artery and its branches and the pulmonary veins and their relationship with the left atrium. These investigations help classify the deformity and choose the appropriate option to repair. Management options include surgical closure of the fistula or device closure using cardiac catheterization.^5^, ^6^, ^7^

We present here a successful surgical closure of a right pulmonary artery to left atrium fistula using a biological patch in a child presenting with central cyanosis.

## 2 CASE

We present a case of a 3.5‐year‐old girl who complained of central cyanosis since birth. Her mother reported exertional fatigue and recurrent respiratory infections. Based on her central cyanosis since birth, the differential diagnosis in this girl includes the following: congenital cyanotic heart diseases as DTGA (D‐transposition of great arteries), TOF (tetralogy of fallot), pulmonary atresia, single ventricle, and truncus arteriosus. Also, chest diseases including pulmonary arteriovenous fistulas and hemoglobin disorders like methemoglobinemia should be considered.

On clinical examination, she had severe cyanosis with grade Ⅲ clubbing in both fingers and toes, and resting oxygen saturation was 74% in room air. Her weight was 13 Kg, and her height was 89 cm. On cardiovascular examination, we found normal heart sounds with a mild systolic heart murmur on the left sternal border. Heart rate was 110 beats/minute. Pulse was palpable and symmetrical in the four limbs.

Pectus excavatum was appreciated on chest examination. The examination of the nervous system was normal.

The mental growth development was appropriate for the child's age. We mention some developmental milestones observed in this child: Language Milestones (Can say her name and age and Speak in sentences of 5‐6 words), Cognitive Milestones (Can correctly name familiar colors and count), Movement Milestones (Can walk up and down stairs, walk forward and backward easily, help put on, and remove clothing), and emotional and social Milestones (Can imitate parents and friends, Understands the idea of "mine" and "his/hers").

The child had frequent respiratory infections and a hospital admission for pneumonia 1 year ago. Her complete blood count (CBC) showed erythrocytosis with hemoglobin concentration of 18 g/dL. ECG (electrocardiography) showed normal sinus rhythm with no abnormalities. CXR (chest X‐ray) showed increased vascularity with an abnormal paracardiac shadow in the right lung.

### 2.1 Echocardiography

We found a secondary ASD 6.5 mm in diameter with a left to right shunt and enlargement of the main pulmonary trunk and the right pulmonary artery. The rest of the echo was normal.

Contrast echo confirmed the abnormal connection between the pulmonary arteries and the left atrium through the visualization of bubbles in the left atrium.

**FIGURE 1:**
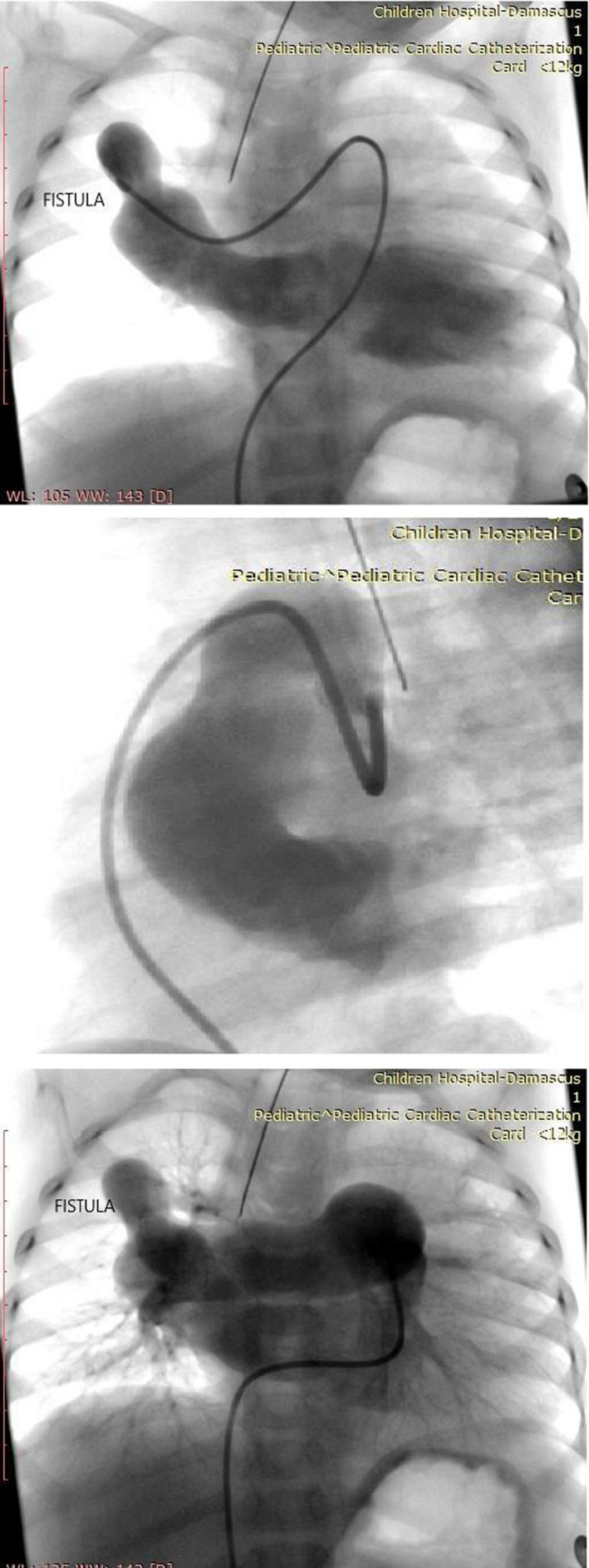
Diagnosis of the fistula between the right pulmonary artery and the left atrium using angiography catheterization

Cardiac catheterization was performed, where the presence of the fistula was confirmed between the right pulmonary artery and the left atrium (Figure 1).

**FIGURE 2:**
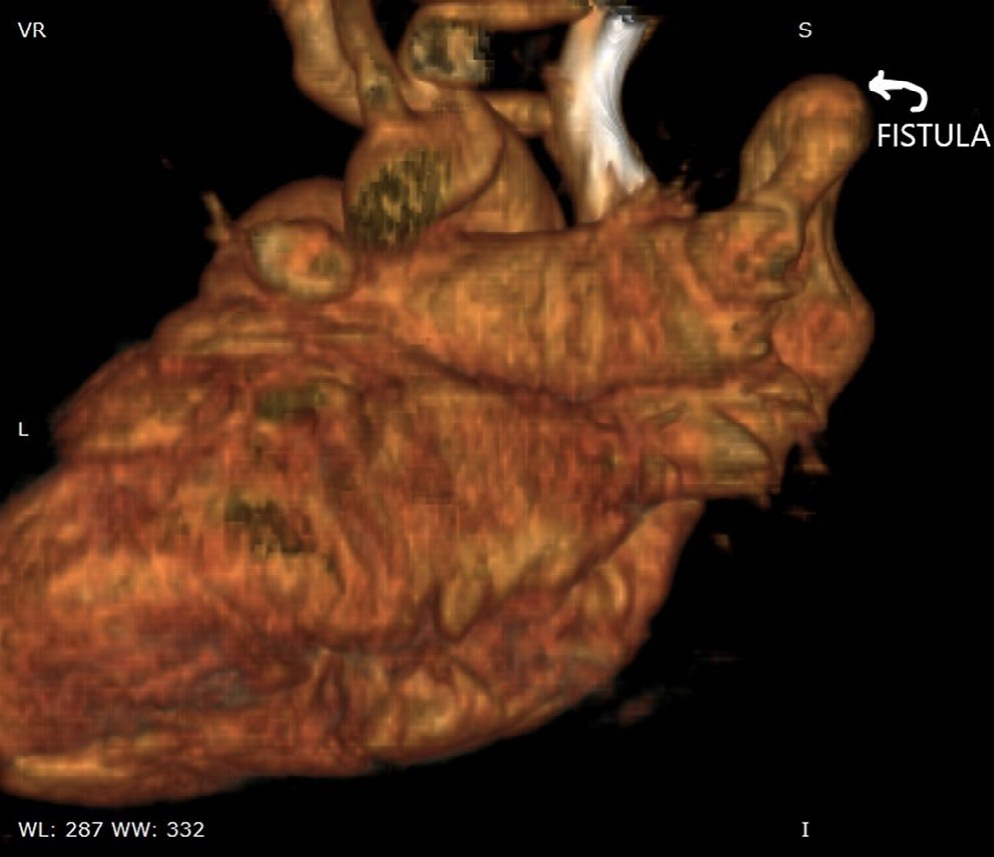
Diagnosis of the fistula between the right pulmonary artery and the left atrium using multislice CT showing the anatomy of the pulmonary vasculature and its relationship with the left atrium through 3D reconstruction

Multislice CT shows that the pulmonary artery branch supplying the middle right lobe drains directly into the left atrium through a large fistula taking the form of a large aneurysm accompanied by an absence of the upper right pulmonary vein (Figure 2).

**FIGURE 3:**
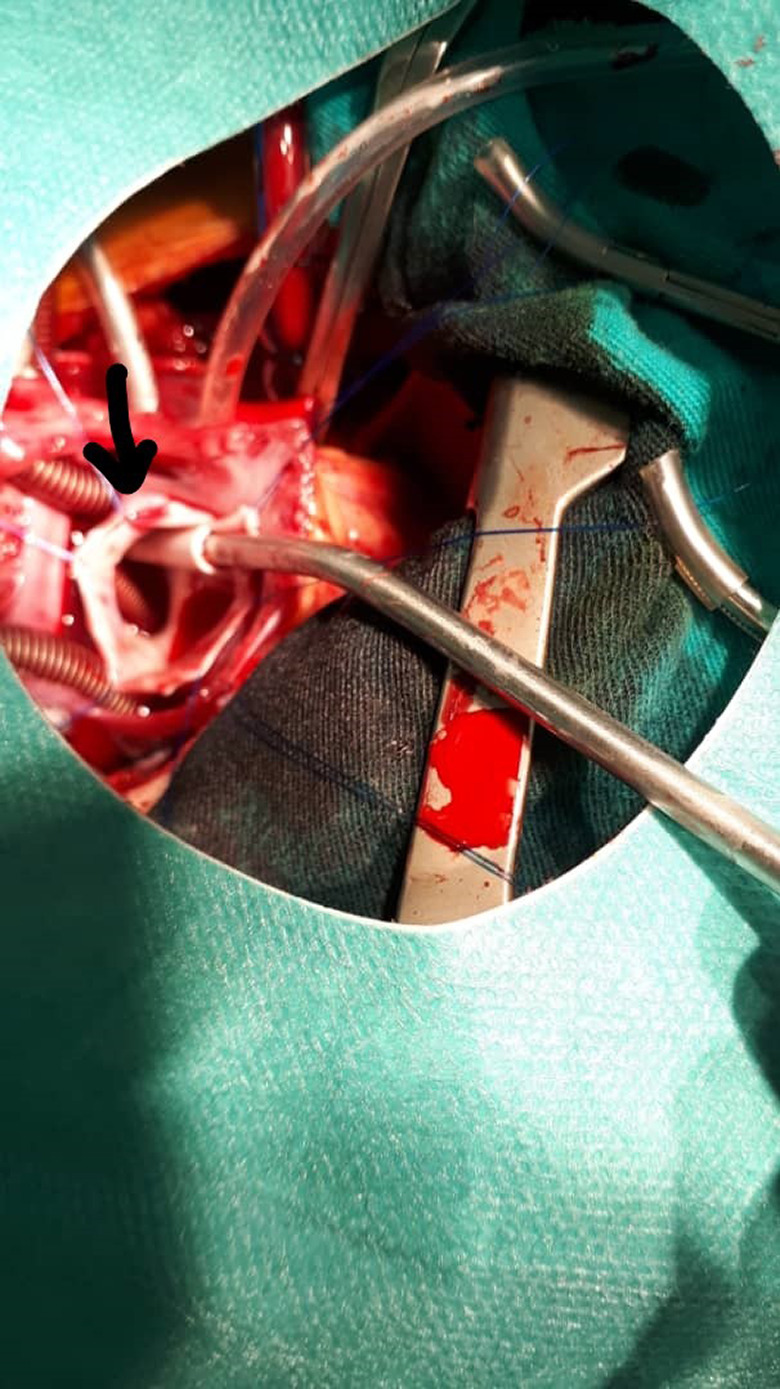
Orifice of the fistula

The surgery was performed under general anesthesia through a median sternotomy. Cardiopulmonary bypass was established, and following cardioplegic arrest, 32˚C blood‐body temperature was provided. Following right atriotomy, the atrial septal defect was found measuring 7 mm, and it was expanded and its edges hung. Through the ASD, the fistula's orifice was seen to measure 20‐25 mm connecting the right pulmonary artery and the left atrium at the point where the right upper pulmonary vein usually opens into the atrium, and the right upper pulmonary vein was absent; then, the fistula's ostium was closed by means of a biological patch using a continuous suture technique with 5/0 prolene. Afterward, the ASD was closed by a biological patch using a continuous suture technique with 5/0 prolene. Other than the ASD, no other shunts or malformations were found during the surgery. (Figure 3).

Oxygen saturation improved to 95% immediately after surgery. On follow‐up after 6 months, the patient was asymptomatic and the clinical examination was normal. (Figure 3).

## 3 DISCUSSION

The clinical findings associated with the presence of a direct fistula between the right pulmonary artery and the left atrium vary from severe to extreme cyanosis with clubbing and exertional dyspnea. These patients experience increased blood viscosity due to the high hemoglobin caused by chronic hypoxia. In addition to this and because of bypassing the pulmonary vascular bed, the blood filtering function of the lung is lost, which leads to complications such as emboli, brain abscesses, cerebrovascular events, and other systemic embolic events,^1^ one of the rare complications that may occur is fistula rupture.

De Souza e Silva et al^8^ described three patterns of fistulae between the pulmonary artery and the left atrium depending on where the fistula opened on the left atrium and its relationship with the branches of the right pulmonary artery and pulmonary veins.

### 3.1 Type 1

The right pulmonary artery and its branches and the pulmonary veins are normal but there is a fistula between the right pulmonary artery and the left atrium.

### 3.2 Type 2

The lower right pulmonary vein is absent, and the lower branch of the right pulmonary artery drains directly to the left atrium where the lower right pulmonary vein usually opens into the atrium.

### 3.3 Type 3

All pulmonary veins connected to the aneurysmal sac between the left atrium and the right pulmonary artery (a fistula).

**FIGURE 4:**
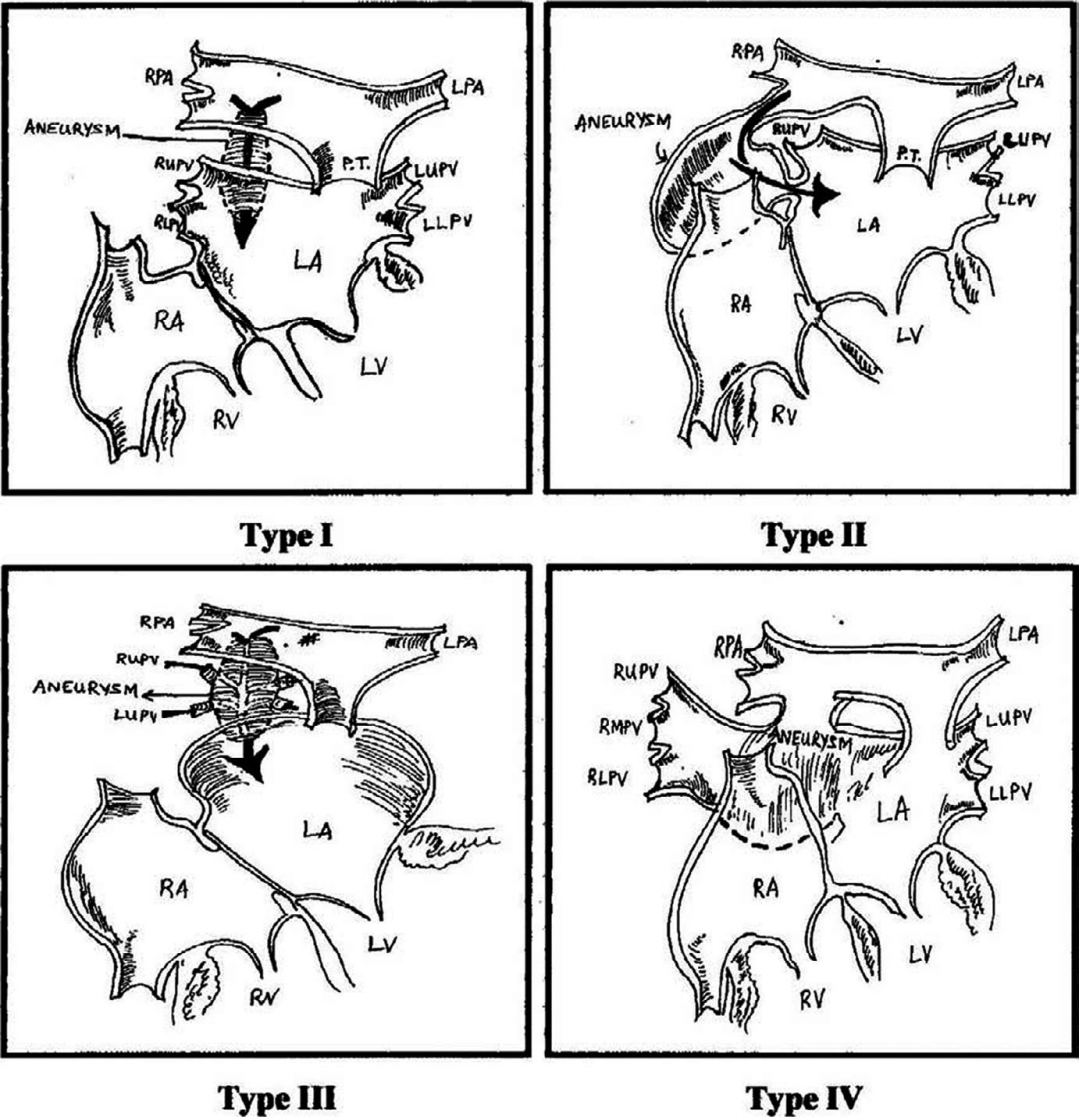
Classification of the fistula between the right pulmonary artery and the left atrium. (MPA main pulmonary artery, RPA right pulmonary artery, LPA left pulmonary artery, LUPV left upper pulmonary vein, LLPV left lower pulmonary vein, RUPV right upper pulmonary vein, RLPV right lower pulmonary vein LA left atrium, RA right atrium, LV left ventricular, RV right ventricular)

Later, the fourth type was added by Ohara et al^9^ This pattern is similar to the second pattern, where right inferior pulmonary vein is replaced by three small veins connected to the aneurysmal sac. (Figure 4).

In the case we present here, the right pulmonary artery which supplies the middle pulmonary lobe drains directly into the left atrium through a large fistula taking the form of an aneurysm where the upper right pulmonary vein usually drains into the atrium.

**FIGURE 5:**
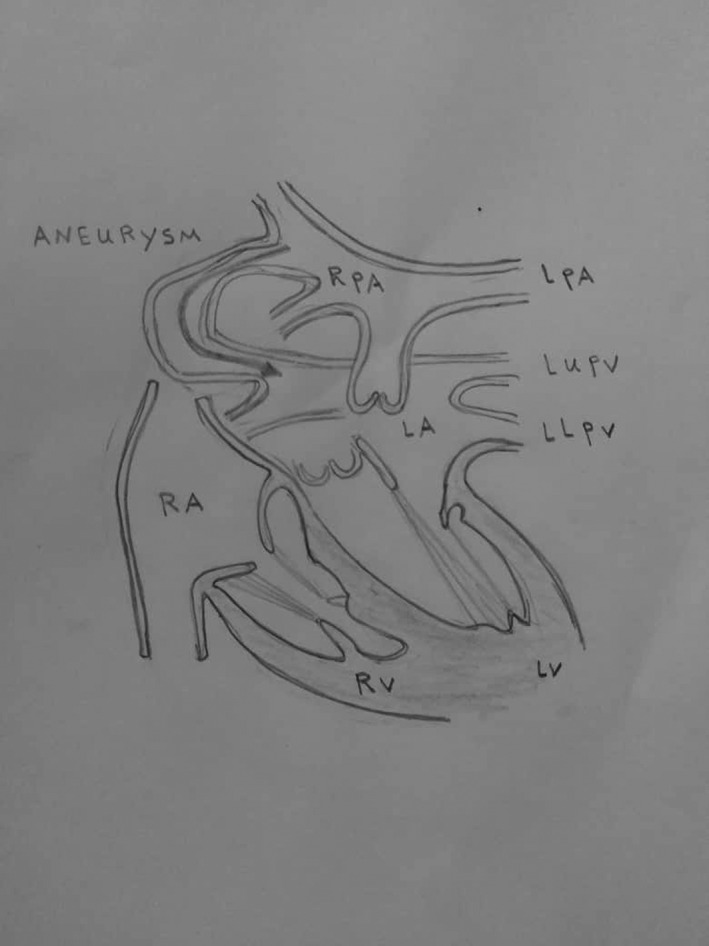
An illustration demonstrating the fistula between the right pulmonary artery and the left atrium mentioned in this case (RPA right pulmonary artery, LPA left pulmonary artery, LUPV left upper pulmonary vein, LLPV left lower pulmonary vein, LA left atrium, RA right atrium, LV left ventricular, RV right ventricular)

This case does not fall under any particular classification but is somewhat similar to Type‐2. (Figure 5).

Embryologically, there is no definite fetal origin or explanation for the fistula between the right pulmonary artery and the left atrium.^10^, ^11^

Atrial septal defect is the most common cardiac defect associated with the fistula between the right pulmonary artery and the left atrium, and the absence of the lower pulmonary lobe and right bronchial atresia are the most common pulmonary malformations.^12^, ^13^

In this case, we found an ASD of a 6.5 mm diameter that was closed at the surgical repair of the fistula.

When diagnosed, this condition should be treated with selective surgery to close the fistula.^14^ This can be achieved either through outside ligation without cardiopulmonary bypass or through open heart surgery using a patch which was performed in the case we presented.

The surgical approach was selected due to the presence of the ASD and the size of the fistula and the thinness of its wall causing concern of rupture.

Many other cases are mentioned in the medical literature regarding the closure of the fistula using cardiac catheterization which is an effective alternative to surgical closure.

## 4 CONCLUSION

In this case, a right pulmonary artery to left atrium fistula was diagnosed as the cause of unexplained cyanosis in a 3.5‐year‐old child. These fistulae are rare cause of cyanosis and require early diagnosis and treatment. The diagnosis was confirmed using cardiac angiography through cardiac catheterization, and the underlying anatomy of the pulmonary vasculature and its relationship with the left atrium was visualized using multislice CT.

This case is significant because of the classification of the fistula does not correspond to the classifications mentioned previously in the medical literature. Surgical repair was performed after diagnosis to prevent serious complications of the fistula.

The patient's oxygenation and symptoms improved immediately after surgery and persisted on follow‐up after 6 months.

## CONFLICT OF INTEREST

None declared.

## AUTHORS CONTRIBUTIONS

AH: analyzed the data, wrote the paper, and edited and supervised the manuscript. Prof. MY: performed the surgery. Prof. SS: diagnosed the patient. MS and MI: edited and prepared the figures and language editing. Dr AH, Prof. MY, and Prof. SS: were equally contributed as first author. Furthermore, all authors discussed the results and commented on the manuscript.

## ETHICAL APPROVAL

The patient provided consent for the publication of this report.
